# Implications of salinity normalization of seawater total alkalinity in coral reef metabolism studies

**DOI:** 10.1371/journal.pone.0261210

**Published:** 2021-12-29

**Authors:** Travis A. Courtney, Tyler Cyronak, Alyssa J. Griffin, Andreas J. Andersson

**Affiliations:** 1 Scripps Institution of Oceanography, University of California San Diego, La Jolla, California, United States of America; 2 Department of Marine Sciences, University of Puerto Rico Mayagüez, Mayagüez, Puerto Rico, United States of America; 3 Department of Marine and Environmental Sciences, Nova Southeastern University, Fort Lauderdale, Florida, United States of America; 4 Bodega Marine Laboratory, University of California Davis, Davis, CA, United States of America; Universite Libre de Bruxelles, BELGIUM

## Abstract

Salinity normalization of total alkalinity (TA) and dissolved inorganic carbon (DIC) data is commonly used to account for conservative mixing processes when inferring net metabolic modification of seawater by coral reefs. Salinity (S), TA, and DIC can be accurately and precisely measured, but salinity normalization of TA (nTA) and DIC (nDIC) can generate considerable and unrecognized uncertainties in coral reef metabolic rate estimates. While salinity normalization errors apply to nTA, nDIC, and other ions of interest in coral reefs, here, we focus on nTA due to its application as a proxy for net coral reef calcification and the importance for reefs to maintain calcium carbonate production under environmental change. We used global datasets of coral reef TA, S, and modeled groundwater discharge to assess the effect of different volumetric ratios of multiple freshwater TA inputs (i.e., groundwater, river, surface runoff, and precipitation) on nTA. Coral reef freshwater endmember TA ranged from -2 up to 3032 μmol/kg in hypothetical reef locations with freshwater inputs dominated by riverine, surface runoff, or precipitation mixing with groundwater. The upper bound of freshwater TA in these scenarios can result in an uncertainty in reef TA of up to 90 μmol/kg per unit S normalization if the freshwater endmember is erroneously assumed to have 0 μmol/kg alkalinity. The uncertainty associated with S normalization can, under some circumstances, even shift the interpretation of whether reefs are net calcifying to net dissolving, or vice versa. Moreover, the choice of reference salinity for normalization implicitly makes assumptions about whether biogeochemical processes occur before or after mixing between different water masses, which can add uncertainties of ±1.4% nTA per unit S normalization. Additional considerations in identifying potential freshwater sources of TA and their relative volumetric impact on seawater are required to reduce uncertainties associated with S normalization of coral reef carbonate chemistry data in some environments. However, at a minimum, researchers should minimize the range of salinities over which the normalization is applied, precisely measure salinity, and normalize TA values to a carefully selected reference salinity that takes local factors into account.

## Introduction

Coral reef metabolic measurements are important tools used to quantify a reef’s carbon cycle, health and function, and responses to ongoing environmental change. Net reef metabolism (e.g. primary production, respiration, calcification, and CaCO_3_ dissolution) is typically quantified through changes in seawater dissolved inorganic carbon (DIC) and total alkalinity (TA) concentrations to determine net ecosystem production (NEP = primary production—total respiration) and net ecosystem calcification (NEC = calcification—CaCO_3_ dissolution) [1, 2]. Notably, positive coral reef net calcification (i.e., +NEC) is critical for maintaining the calcium carbonate structures and the associated ecosystem services that coral reefs provide [3]. Herein, we evaluate the salinity normalization of TA data and its impact on using alkalinity as a proxy for evaluating coral reef net calcification.

The total alkalinity anomaly technique is pragmatic for determining NEC in coral reefs because TA changes by a factor of two for every unit of CaCO_3_ formed or dissolved [4–6] with negligible to little influence from other processes (e.g., uptake and release of nutrients) in most coral reef environments [7]. Furthermore, TA is conservative with respect to mixing and changes in seawater temperature and pressure [8]. As a result of reef calcification, TA can vary by upwards of hundreds of μmol/kg across coral reef environments [2]. Consequently, the alkalinity anomaly method, which calculates the difference between initial (e.g., offshore, upstream, or proximal) and final (e.g., coral reef, downstream, or distal) TA can be used to determine whether a reef is net calcifying and combined with measurements of depth and water residence time or transit times to quantify rates of coral reef net ecosystem calcification [5, 9]. However, to account for any changes in TA due to freshwater dilution, evaporation, and mixing between water masses, TA data are commonly normalized with respect to changes in salinity, resulting in a salinity normalized alkalinity anomaly (i.e., ΔnTA). Throughout this manuscript, we will use the notation ΔnTA = nTA_offshore_–nTA_reef_ to maintain consistency, but it is important to note that ΔnTA could represent any nTA_initial_–nTA_final_. Salinity normalization of TA is performed using a reference salinity (S_ref_), freshwater TA endmember (TA_S = 0_), and salinity (S) data per the following equation [10]:

$$nTA=\frac{TA-TA_{S=0}}{S}\times S_{ref}+TA_{S=0}$$ (1)

**Fig 1:**
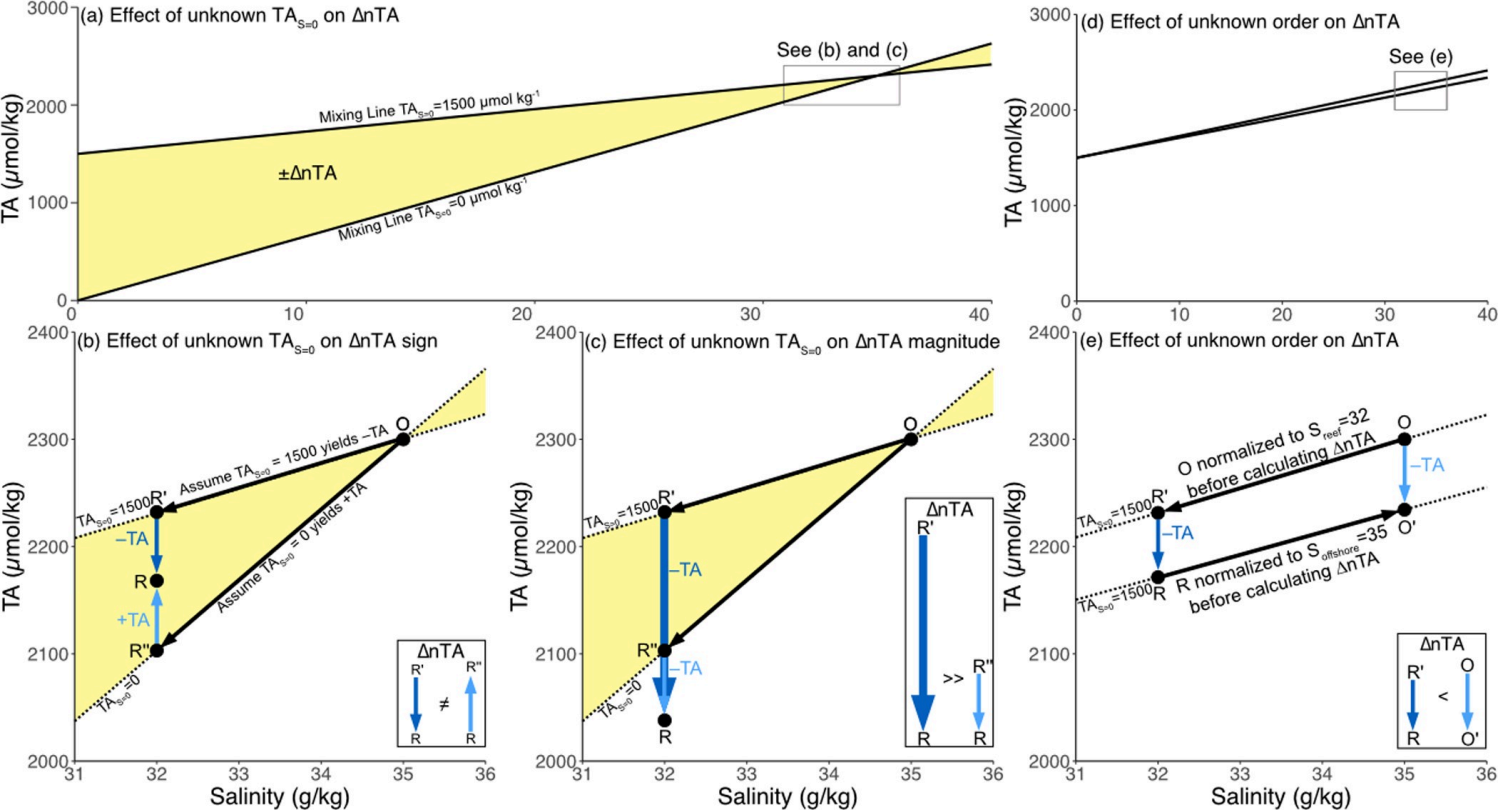
Conceptual diagram of salinity normalization of coral reef total alkalinity data. (a,b,c) Conceptual diagram demonstrates how conservative mixing of a representative surface TA-S of open ocean (i.e., designated by O on plot; TA_offshore_ = 2300 μmol/kg, S_offshore_ = 35 g/kg) with a representative freshwater TA endmember of TA_S = 0_ = 1500 (e.g., see simulations below) or TA_S = 0_ = 0 can create considerable uncertainties in the (b) sign and (c) magnitude of ΔnTA calculated with respect to the coral reef seawater TA (i.e., TA_reef_ designated by R on plot). The yellow regions indicate the uncertainty of ΔnTA if the actual TA_S = 0_ is unknown but within two values in this region. The ΔnTA box (b, c) shows the magnitude of uncertainty for ΔnTA between the ocean (O) and reef (R) owing to an unknown TA_S = 0_ where ΔnTA is not equal (≠) in direction in (b) and ΔnTA is much greater (>>) when assuming TA_S = 0_ = 1500 μmol/kg compared to TA_S = 0_ = 0 μmol/kg in (c). Conceptual diagram demonstrates how assuming mixing occurs 1^st^ (S_ref_ = S_reef_) or calcification occurs 1^st^ (S_ref_ = S_offshore_) affects the magnitude of ΔnTA inferred from salinity normalized changes in coral reef TA. ΔnTA box shows the magnitude of uncertainty for ΔnTA owing to biogeochemical processes at point R relative to point O such that ΔnTA = R’–R is less than (<) ΔnTA = O–O’. The conceptual framework is expanded from Fig 9 in [16] to show how unknown TA_S = 0_ (a,b,c) and unknown order of processes (d,e) can affect interpretation of the magnitude and direction of coral reef NEC determined from changes in seawater total alkalinity.

Salinity normalization of seawater TA in coral reef metabolism studies has traditionally used simple dilution concentration (SDC) mechanisms (i.e., freshwater dilution and evaporation) between TA_offshore_ and TA_S = 0_ = 0 (Eq 1) [10]. However, assumptions regarding SDC can be invalidated by processes that modify freshwater alkalinity such as the weathering of carbonate and silicate minerals [11, 12], anaerobic redox processes, and the anthropogenic deposition of sulfuric and nitric oxides as acid rain [13, 14]. Coral reefs are complex coastal ecosystems with many potential sources of freshwater TA (i.e., TA_S = 0_) including groundwater (95–13,000 μmol/L [15]), tropical rivers (224–2,156 μmol/L [12]), and precipitation (-2.7–18 μmol/L [14]) such that:

$$TA_{S=0}=\chi_{gw}TA_{gw}+\chi_{rw}TA_{rw}+\chi_{p}TA_{p}$$ (2)

where χ represents the relative volumetric freshwater proportion of groundwater (χ_gw_), riverine water (χ_rw_), or precipitation (χ_p_), and their TA concentrations, respectively. If freshwater sources with significant TA_S = 0_ are volumetrically important, but their concentrations and relative proportions are unknown (Eq 2), the system is underdetermined and could result in errors in both the magnitude and direction of coral reef ΔnTA (Fig 1A–1C). Moreover, in coastal ecosystems such as coral reefs, it is challenging to accurately estimate freshwater alkalinity endmembers (TA_S = 0_), and consequently nTA, from TA-S relationships because evaporation and net calcification further modify TA-S mixing lines [16]. The presence of multiple potential freshwater TA sources and variable mixing ratios co-occurring with biogeochemical modifications of TA raise concerns that unknown TA_S = 0_ could be a source of significant uncertainty in coral reef ΔnTA estimates and, consequently, estimates of net coral reef calcification rates.

In addition to the effects of unknown TA_S = 0_ on ΔnTA, the application of salinity normalization and the choice of a reference salinity implicitly introduce assumptions about whether conservative mixing occurs before biogeochemical modification (i.e., net ecosystem calcification) or vice versa [16]. For example, Fig 1D and 1E illustrate mixing and biogeochemical modification between offshore TA-S (point O) and reef TA-S (point R). By salinity normalizing coral reef TA (point R) to open ocean endmember salinity (point O) before calculating ΔnTA, R is normalized to O’ and ΔnTA is calculated as the difference in TA between O and O’ (Fig 1D and 1E). This scenario (S_ref_ = S_offshore_) implicitly assumes that calcification occurs prior to mixing with the freshwater endmember. Conversely, by salinity normalizing open ocean TA endmember (point O) to the coral reef salinity (point R) before calculating ΔnTA, O is normalized to R’ and ΔnTA is calculated as the difference in TA between R’ and R (Fig 1E). This scenario (S_ref_ = S_reef_) implicitly assumes that mixing with the freshwater endmember occurs prior to calcification. The ΔnTA inferred from O and O’ is slightly greater than the ΔnTA inferred from R’ and R (Fig 1E). Therefore, the choice of S_ref_ in the salinity normalization of coral reef TA data via Eq 1 implicitly assumes whether calcification occurs before mixing (i.e., S_ref_ = S_offshore_), mixing occurs before calcification (i.e., S_ref_ = S_reef_), or some order of mixing between these two extremes (i.e., S_reef_<S_ref_<S_offshore_) and generates an additional source of uncertainty in coral reef ΔnTA.

In a previous study, we suggested that uncertainties in seawater depth and residence time can potentially drive large uncertainties in estimates of coral reef NEC rates while assuming that the accurate determination of TA (±2 μmol/kg) was unlikely to be a significant source of error [17]. Here, we quantify the potential uncertainties in ΔnTA associated with the salinity normalization of coral reef total alkalinity to unknown freshwater endmembers (TA_S = 0_) and reference salinities (S_ref_) using global seawater TA and S, fresh submarine groundwater discharge, a range of different TA_S = 0_ datasets, and different mixing proportions of freshwater endmembers. We provide a series of suggestions to reduce the uncertainties associated with salinity normalization of coral reef TA data with implications for other carbonate chemistry parameters and ions of interest to coral reef metabolism studies (e.g., DIC, Ca^2+^, Mg^2+^, etc.).

## Methods

### Estimating potential coral reef TA_S = 0_

**Fig 2:**
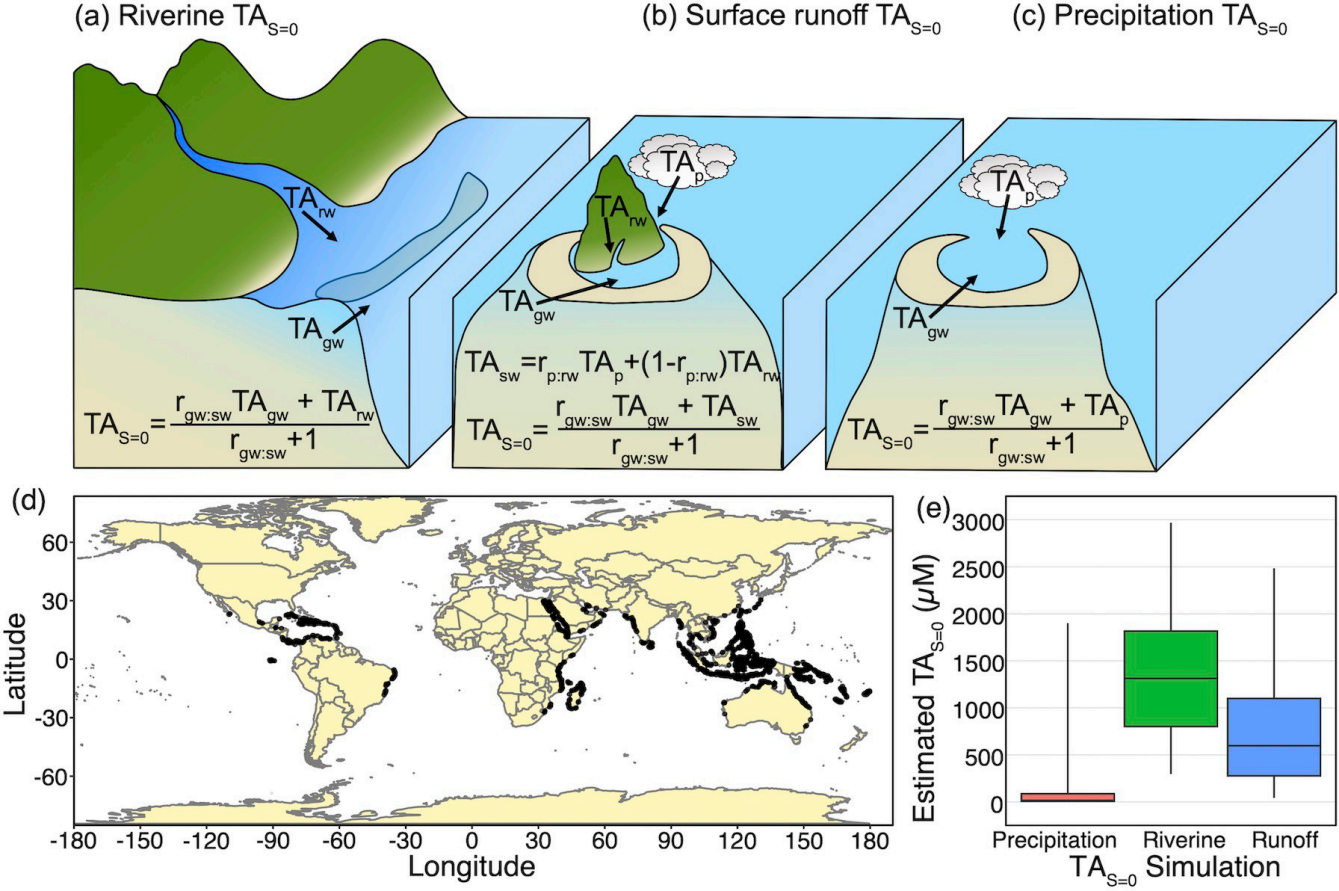
Simulations of freshwater total alkalinity endmembers. (a,b,c) Conceptual diagram shows the relative contributions of freshwater TA endmembers (i.e., TA_S = 0_) for simulations of (a) riverine dominated, (b) surface runoff dominated, and (c) precipitation dominated reef systems where r_gw:sw_ = ratio of groundwater to surface water, r_p:rw_ = ratio of precipitation to riverine water, TA_gw_ = groundwater TA, TA_rw_ = riverine TA, TA_p_ = precipitation TA, and TA_sw_ = surface water TA. (d) Map shows the coral reef locations with fresh submarine groundwater discharge in [18] used with the equations in (a, b, c) to calculate (e) coral reef TA_S = 0_ box plots representing 2.5^th^, 25^th^, 50^th^, 75^th^, and 97.5^th^ percentiles of estimated coral reef TA_S = 0_ from the Monte-Carlo simulations for the precipitation, riverine, and surface runoff dominated reef systems.

In the absence of detailed freshwater budgets and relative volumetric proportions of different freshwater TA endmembers in coral reefs, we simulated coral reef TA_S = 0_ using literature derived data in a Monte Carlo approach (n = 10,000) assuming three distinct scenarios: (i) riverine + groundwater freshwater inputs (Fig 2A); (ii) terrestrial surface runoff + groundwater freshwater inputs (Fig 2B); and (iii) precipitation + groundwater freshwater inputs (Fig 2C). For each scenario, a random selection was made from the ratio of fresh submarine groundwater discharge to surface runoff for coral reefs (n = 4,241; Fig 2D) calculated by [18]. We then combined this selected ratio of groundwater inputs value with randomly generated TA concentrations from within the range of the typical coral reef groundwaters (95–13,000 μmol/L [15]), tropical rivers (224–2,156 μmol/L [12]), and precipitation (-2.7–18 μmol/L [14]). This analysis allowed us to simulate the potential TA concentration of mixed freshwater endmembers for each of the three scenarios described above (Fig 2A). The simulated coral reef TA_S = 0_ were positively skewed so the 50^th^, 2.5^th^, and 97.5^th^ percentiles were determined for each of three scenarios to approximate the median and range of potential coral reef TA_S = 0_.

### Global coral reef total alkalinity data

We used a global assessment of coral reef seawater carbonate chemistry and salinity data [2] to quantify coral reef TA-S relationships, salinity ranges, and the effect of salinity normalization on TA_reef_. To assess TA-S relationships, type II ordinary least squares linear regression of TA vs. S were constructed for each of the 27 coral reefs using the function *lmodel2* [19] in *R* [20]. To evaluate the range and distribution of salinity changes, we subtracted every salinity value from the maximum salinity value for each of the 27 coral reefs. nTA_reef_ was calculated via Eq (1) using the mean salinity for each site (i.e., S_ref_ = S_mean_) and assuming TA_S = 0_ = 0. We then calculated the difference between TA_reef_ and nTA_reef_ for each of the 27 coral reefs to estimate the magnitude to which salinity normalization changes TA_reef_ data.

### Effects of unknown TA_S = 0_ on ΔnTA

We quantified nTA_reef_ via Eq (1) using the maximum salinity for each site as the reference salinity (i.e., S_ref_ = S_max_) and TA_S = 0_ values of 0, 1000, 2000, and 3000 μmol/kg, which span the full range of potential simulated freshwater TA endmembers in coral reef ecosystems from this study (Fig 2E). In the absence of reported S_offshore_ for every coral reef location, we assumed that S_max_ represents S_offshore_ to calculate ΔnTA as the mean of TA_offshore_–nTA_reef_ and ΔS as the mean of S_max_–S_reef_ for all samples from each reef location in [2]. Furthermore, because the true TA_S = 0_ for each reef location were unknown, we quantified the uncertainties in ΔnTA owing to mixing within the range of freshwater TA endmembers explored in this study. To accomplish this, we subtracted the ΔnTA values calculated assuming a zero freshwater TA endmember from the ΔnTA values calculated assuming a positive freshwater TA endmember (i.e., ΔnTA_TA>0@S = 0_–ΔnTA_TA = 0@S = 0_). Linear regression of these ΔnTA uncertainty vs. ΔS relationships for each TA_S = 0_ represent the potential range (i.e., uncertainty) in the actual ΔnTA values for a given change in S assuming the true TA_S = 0_ for each reef site is between the 0 and 3000 μmol/kg values explored in this analysis. To further explore how the precision of S can affect nTA, the minimum difference in reported salinity data for each reef was used to estimate the precision of the salinity measurement.

### Effects of unknown order of processes on ΔnTA

To quantify how the implicit order of mixing and biogeochemical processes impact coral reef ΔnTA data, we quantified ΔnTA as either ΔnTA_offshore_ = TA_offshore_–nTA_reef_ (i.e., S_ref_ = S_offshore_ assuming calcification occurs before mixing; O to O’ to R sensu Fig 1E) or ΔnTA_reef_ = nTA_offshore_–TA_reef_ (i.e., S_ref_ = mean(S_reef_) assuming mixing occurs before calcification; O to R’ to R sensu Fig 1E) for each reef location in [2]. We then quantified the mean percent difference between ΔnTA_offshore_ and ΔnTA_reef_ for each site to evaluate the type II ordinary least squares linear regression between % ΔnTA uncertainty and ΔS for each reef site using the function *lmodel2* [19] in *R* [20] to see how uncertainty in ΔnTA scales with ΔS owing to the unknown order of mixing and biogeochemical processes.

## Results and discussion

### Coral reef TA_S = 0_

The global ratio of coral reef fresh submarine groundwater discharge to surface water runoff database [18] provided an opportunity to simulate TA_S = 0_ for coral reef locations where precipitation, riverine, and freshwater runoff occur in different volumetric proportions (Fig 2). Coral reef TA_S = 0_ estimates were positively skewed with median (2.5^th^ to 97.5^th^ percentile) values of 15 μmol/L (-2 to 1997 μmol/L) for groundwater mixing with precipitation, 1306 μmol/L (293 to 2979 μmol/L) for groundwater mixing with river water, and 598 μmol/L (43 to 2507 μmol/L) for groundwater mixing with surface runoff (Fig 2E). The range of simulations in this study suggest that these uncertainties vary between coral reefs owing to proximity to terrestrially derived freshwater inputs and over time and space within coral reefs owing to temporally and geographically variable freshwater inputs (Fig 2). In particular, assessing the proximity of the reef system to land masses and/or notable surface freshwater inputs could inform which of the three simulated coral reef TA_S = 0_ (± uncertainty) scenarios explored in this study may best represent uncertainties associated with unknown TA_S = 0_ in future studies where TA_S = 0_ and their respective volumetric proportions are unknown. However, we nonetheless caution that these simulations are hypothetical (Fig 2D) and that TA_S = 0_ may exceed these estimates in select coral reefs with elevated volumetric proportions of groundwater or within individual coral reefs where the assumption of complete mixing between multiple TA_S = 0_ is invalid. Submarine groundwater discharge can be patchy and heterogeneous across small spatial scales, meaning biogeochemical processes and hydrodynamics should be assessed locally [21, 22]. Conversely, the influx of low TA_S = 0_ owing to precipitation falling directly on the reef and/or adjacent lagoon waters could drive mean freshwater TA_S = 0_ lower than the simulated values in this study. Moreover, land-based precipitation is likely to reach the reef in the form of runoff, riverine inputs, and/or groundwater at some time lag following the initial precipitation event, suggesting that the timing of precipitation events is an important consideration in estimating the relative volumetric contributions of multiple TA_S = 0_ sources.

### Global coral reef TA-S relationships

**Fig 3:**
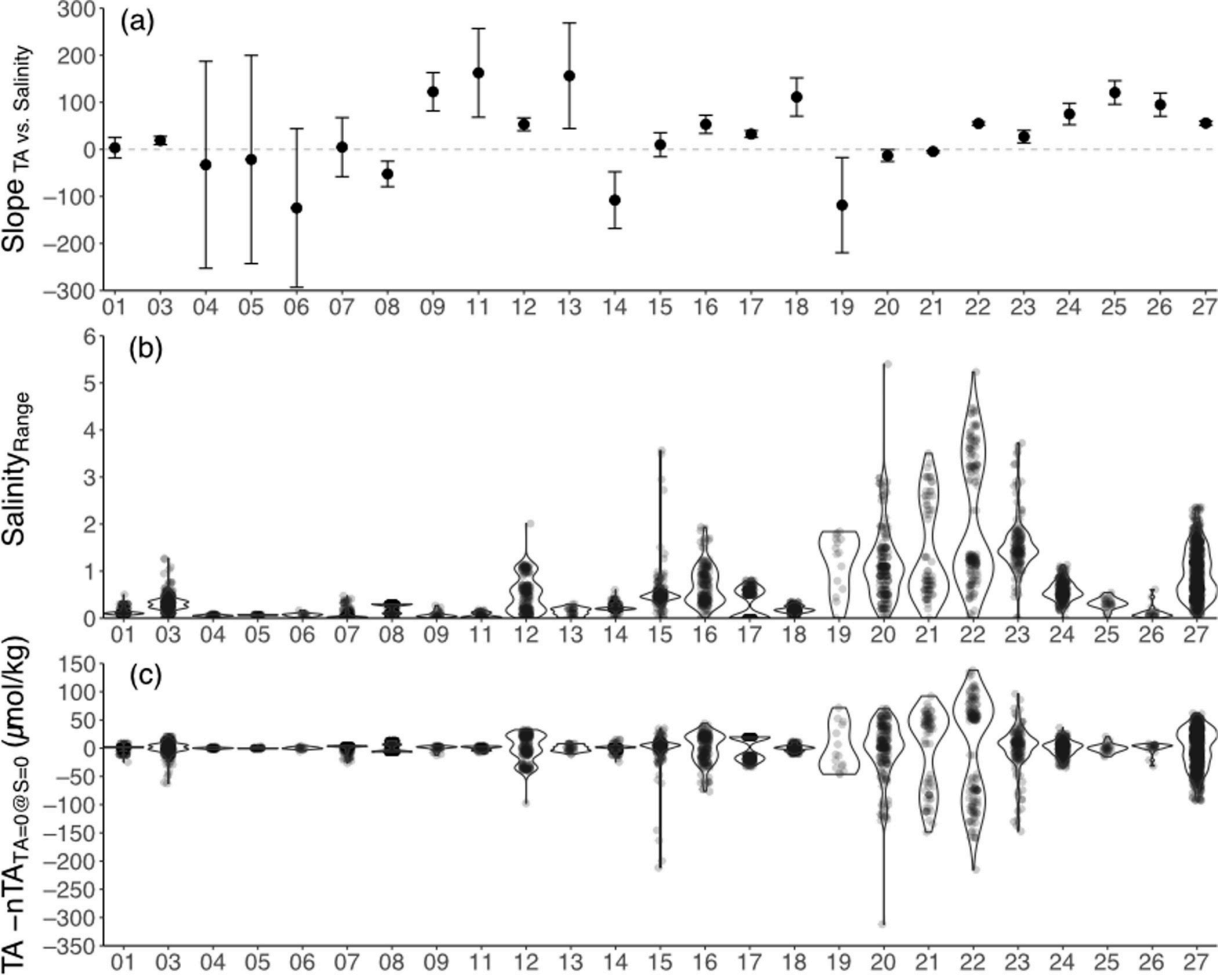
Global coral reef total alkalinity and salinity relationships. Relationships between TA, S, and nTA are explored across global coral reef environments from [2]. (a) The slope (± 95% confidence interval) of linear regressions between TA and S for each reef. Slopes that do not overlap the dashed zero line are detectably different from zero. (b) The range of salinities defined as the S_max_–S for each reef datapoint with all data outlined by the probability density for each site to visualize the distribution. (c) The difference between TA and nTA calculated using S_mean_ as the S_ref_ and TA_S = 0_ = 0 via Eq (1) for each reef with all data outlined by the probability density for each site to visualize the distribution. Sources of coral reef data are 01 = Makapu’u, 03 = Palmyra, 04 = Red Sea 1997, 05 = Red Sea 1998, 06 = Red Sea 2013, 07 = Ofu, 08 = Heron Island, 09 = Palau 1, 11 = Lizard Island, 12 = Ishigaki, 13 = One Tree 1, 14 = Kaiona, 15 = Lady Elliot, 16 = One Tree 2, 17 = Davies, 18 = Palau 2, 19 = Cook Islands, 20 = Cheeca Rocks, 21 = St. John, 22 = West Panama, 23 = Florida Keys, 24 = Bermuda, 25 = Majuro, 26 = Maldives, and 27 = Puerto Rico sensu [2]. See references and discussion in [2] for further details on each coral reef location.

We used the global dataset of TA and S data to explicitly test TA-S relationships in coral reef metabolism studies conducted over a broad range of spatial and temporal scales [2]. The majority of coral reefs exhibited detectable linear relationships between TA and S (i.e., 19 of 25 reefs have 95% confidence intervals that do not overlap the dashed 0 line) (Fig 3A and S1 Table), which indicates the need for salinity normalization of TA_reef_ data to account for mixing of water with different properties and the influence of freshwater inputs or evaporation on TA [10]. Notably, data from two reefs exhibited no reported changes in S so the TA-S slopes were undefined for these reef sites and removed from this analysis (i.e., 02 = Mo’orea and 10 = Yonge Reef). Mean ranges in S_reef_ (i.e., S_max_−S_reef_) for each of the reefs varied from 0.05 to 2.1 (mean ± SD for all reefs = 0.5±0.6) (Fig 3B). Mean (±SD) differences between non-normalized and normalized TA (i.e., assuming TA_S = 0_ = 0 and S_ref_ = S_max_ = S_offshore_ via Eq 1) were -0.2±30.4 μmol/kg (range = -311.9 to +138.2 μmol/kg) (Fig 3C). This mean difference of -0.2 μmol/kg supports previous findings that salinity normalization had a negligible impact on mean ΔnTA across the reef sites presented here [2], especially for reef sites with minimal S ranges (Fig 3B and 3C). However, the range of -311.9 to +138.2 μmol/kg values is the same magnitude as the estimated changes in TA strictly owing to coral reef NEC [17] suggesting that salinity normalization of coral reef TA can nonetheless have a significant impact on individual ΔnTA estimates with greater deviations in salinity from S_ref_.

### Estimated effects of unknown TA_S = 0_ on nTA_reef_

**Fig 4:**
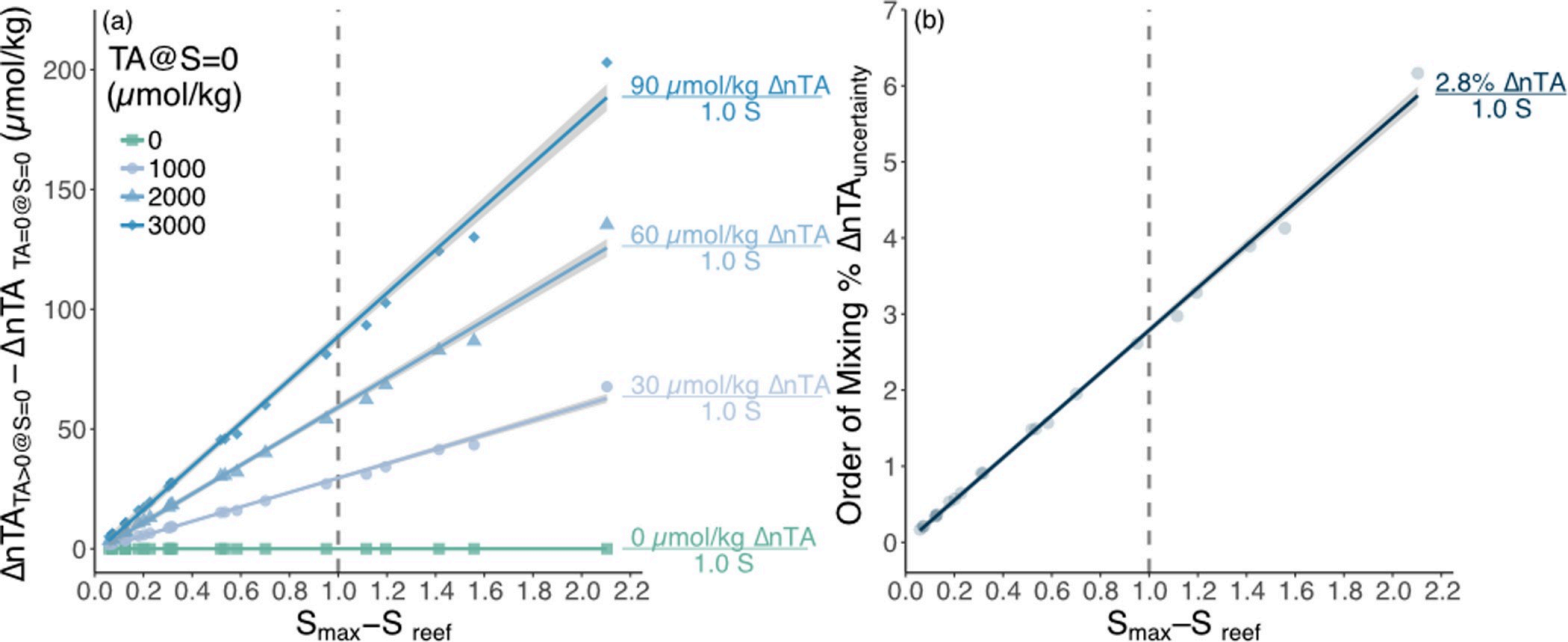
Salinity normalized alkalinity anomaly uncertainties scale with salinity changes. (a) The mean alkalinity anomaly for each reef evaluated for TA_S = 0_ ranging from 0, 1000, 2000, 3000 μmol/kg using S_ref_ = S_max_ in Eq (1) minus the the mean alkalinity anomaly for TA_S = 0_ are plotted against the mean S_max_–S_reef_ for each reef location in [2] to quantify how uncertainty in ΔnTA scales with unknown TA_S = 0_. Values to the right of each line plotted on the graph are the uncertainty in ΔnTA per unit salinity for the respective TA_S = 0_ line. (b) The mean percent difference between ΔnTA_offshore_ and ΔnTA_reef_ owing to the choice of S_ref_ of S_max_ = S_offshore_ or S_reef_ via Eq (1) are plotted against the mean S_max_–S_reef_ for each reef location in [2] to evaluate how uncertainty associated with the order of mixing processes scale with salinity changes across the reef. The vertical dashed gray line indicates uncertainty per unit salinity change in each panel.

The alkalinity anomalies for each reef location normalized to different TA_S = 0_ show the potential for non-negligible uncertainties in ΔnTA if a positive TA_S = 0_ is not accounted for (Fig 4A). Most importantly, the magnitude of the difference between ΔnTA calculated assuming a positive freshwater TA endmember and ΔnTA calculated assuming a zero freshwater TA endmember (i.e., ΔnTA_TA>0@S = 0_ – ΔnTA_TA = 0@S = 0_) scaled with the magnitude of the difference between S_max_ and S_ref_ (Eq 1 and Fig 4A). Maximum uncertainties associated with freshwater (TA_S = 0_) equal to 1000, 2000, and 3000 μmol/kg compared to TA_S = 0_ = 0 μmol/kg were 30, 60, and 90 μmol/kg per unit difference in salinity, respectively (Fig 4A). Assuming that TA_S = 0_ = 0 in the salinity normalization of coral reef TA via Eq 1 can therefore yield uncertainties in ΔnTA that increase linearly with increasing TA_S = 0_. Similarly, if TA_S = 0_>0 is assumed during salinity normalization, then TA uncertainties increase with increasing offset between TA_S = 0_ and the assumed TA_S = 0_. Establishing potential upper and lower bounds on TA_S = 0_ for different freshwater endmembers on coral reefs and their relative volumetric contributions to the reef therefore provide an effective means to reduce the potential range of uncertainties associated with the salinity normalization of TA_reef_ (Fig 2E). The canonical assumption that TA_S = 0_ = 0 for salinity normalization [10] is close to the median TA_S = 0_ = 15 μmol/L for the precipitation mixing with groundwater scenario in this study, which suggests that TA_S = 0_ = 0 may remain a reasonable approximation at least for precipitation dominated reef systems and habitats. However, it is important to note that this is from simulated freshwater endmembers in coral reef ecosystems and freshwater TA concentrations can vary considerably in reef ecosystems. Calculating nTA using the full range of TA_S = 0_ for precipitation dominated systems (i.e., -2 to 2038 μmol/L) may nonetheless provide a more honest assessment of nTA ± uncertainties for cases where TA_S = 0_ and the relative volumetric freshwater fluxes are unknown.

We recommend viewing coral reefs from an ecosystem-scale perspective to consider the volumetric input of freshwater from different sources (i.e., groundwater, river input, and/or runoff account for a substantial fraction of freshwater input) when using ΔTA as a proxy for coral reef calcification and, if warranted, locate and directly measure TA_S = 0_ of these freshwater endmembers. However, the multitude of endmembers and biogeochemical processes increasingly complicate estimates of the mixing derived component of coral reef TA_reef_:S variability. While the estimated freshwater TA endmembers for precipitation, riverine, and runoff dominated systems from this study (Fig 2E) may provide alternative TA_S = 0_ estimates ± uncertainties wherever the precise quantification of TA_S = 0_ values and relative volumetric inputs is not feasible, any assumptions regarding the salinity normalization of TA data should nonetheless be carefully considered (e.g., see below list of considerations). Moreover, utilizing other tracers to normalize seawater TA data such as silicate, Ra, Rn, Li, Mg, Sr, Ca, Ba, δ^18^O, and other tracers for submarine groundwater discharge [22–25] warrant further consideration as additional means to parse out the relative contributions of mixing of multiple freshwater endmembers and biogeochemical fluxes on coral reef seawater carbonate chemistry data.

### Effects of unknown order of processes on ΔnTA

The ΔnTA uncertainties owing to the implicitly assumed order of mixing and biogeochemical processes in the salinity normalization of TA were much smaller than the uncertainties owing to the unknown TA_S = 0_ (Fig 4). For example, there was a 2.8% range of uncertainty in ΔnTA per unit salinity owing to normalizing ocean and coral reef TA data to S_offshore_ compared to S_reef_ (Fig 4B). Assuming an actual 100 μmol/kg alkalinity anomaly solely owing to net calcification, this 2.8% uncertainty per unit salinity translates to a 2.8 μmol/kg uncertainty range, which is similar to the typical precision of ±2 μmol/kg for coral reef TA measurements [17]. The choice of normalizing to S_offshore_ or S_reef_ represent extreme scenarios where either all calcification is assumed to occur before all mixing (S_ref_ = S_offshore_) or all mixing is assumed to occur before all calcification (S_ref_ = S_reef_) (Fig 1D and 1E). Unless there is information to rigorously evaluate which order of processes is occuring, calculating both ΔnTA_offshore_ using S_ref_ = S_offshore_ (i.e., calcification then mixing) and ΔnTA_reef_ using S_ref_ = S_reef_ (i.e., mixing then calcification) quantifies the maximum uncertainty associated with the unknown order of mixing and calcification processes as follows:

$$\Delta nTA_{offshore}=TA_{offshore}-\left[\left(TA_{reef}-TA_{S=0}\right)\frac{s_{offshore}}{s_{reef}}+TA_{S=0}\right]$$ (3)

$$\Delta nTA_{reef}=\left[\left(TA_{offshore}-TA_{S=0}\right)\frac{S_{reef}}{S_{offshore}}+TA_{S=0}\right]-TA_{reef}$$ (4)

Importantly, the average of ΔnTA_offshore_ and ΔnTA_reef_ results in a ΔnTA that assumes net calcification and mixing occur simultaneously and results in a centered, quantifiable ±1.4% uncertainty in ΔnTA per unit salinity change.

### Salinity and TA_reef_ variability

In studies aimed at assessing net reef calcification, the simplest way to reduce uncertainties in coral reef nTA is by selecting sampling sites and time periods with minimal S variability (Figs 3 and 4). Selecting sites with known hydrodynamics could further reduce mixing related uncertainties. Any deviations between S_offshore_ and S_reef_ have the potential to generate non-negligible uncertainties in coral reef salinity normalized total alkalinity anomalies (i.e., ΔnTA = nTA_offshore_–nTA_reef_). While we have assumed that S_max_ = S_offshore_ to fill in missing data from [2], S_offshore_ should ideally be carefully quantified alongside TA_offshore_ to reduce actual ΔnTA uncertainties in subsequent studies. Critically, the choice of sampling location for TA_offshore_ represents an important consideration depending on the aims of the study and characteristics of the coral reef being investigated. For example, choosing an offshore reference that is more proximal to the reef environment can minimize the difference between S_offshore_ and S_reef_ to reduce uncertainties owing to a large range in salinity. Depending on the specifics of individual reef systems and nearshore oceanographic processes, TA_offshore_ of these more proximal offshore waters may be more strongly influenced by other nearshore processes such as upwelling and coastal freshwater inputs [16]. As a result, the choice of sampling location for offshore reference samples is likely context dependent with the capacity to impact both the salinity normalization of TA and the relative contributions of non-reef processes on ΔnTA.

While TA is often evaluated with a high degree of precision and accuracy (i.e., many labs were within ±2 μmol/kg and the majority of labs were within ±10 μmol/kg in a recent intercomparision study; [26]), the estimated precision of reported salinity measurements were ≤0.01 for 18 reefs, 0.03 for 1 reef, 0.1 for 6 reefs, and undefined for the two reef sites which reported no changes in S. Even small salinity changes of ≤0.1 across samples can drive significant uncertainties in ΔnTA suggesting that ideally the most precise and accurate S measurement should be used in the evaluation of coral reef ΔnTA. For example, the rounding of 34.44 to 34.4 and 34.45 to 34.5 by a salinity precision of ±0.1 shows how a real 0.01 salinity change can result in an apparent 0.1 salinity change. In this case, normalizing TA to an apparent salinity change of ±0.1 could generate uncertainties up to 9 μmol/kg in nTA values based on the TA_S = 0_ = 3000 μmol/kg relative to TA_S = 0_ = 0 μmol/kg scenario. With current salinity sensing technology, uncertainties in salinity and, consequently, nTA could be much smaller. Therefore, any such studies that salinity normalize their TA data may be underreporting the true uncertainties in their measurements by simply stating the precision of their TA measurements and not including the uncertainties owing to their S measurements and subsequent salinity normalization. Furthermore, if changes in salinity are within the reported uncertainties of the salinity measurement, then there is not sufficient evidence that salinity has detectably changed across the reef environment. In these cases, salinity normalization may introduce unnecessary error into TA changes, and it may be more appropriate to avoid salinity normalization if the S_range_ is within the reported precision and/or accuracy of the salinity measurement.

### Implications for coral reef net ecosystem calcification

Here we have shown that the uncertainty of ΔnTA measurements is likely greater than the reported ± 2 to 10 μmol/kg precision of TA owing to uncertainties associated with the normalization of TA_reef_ data to S_ref_ and especially if TA_S = 0_ is unknown. We have further provided quantitative simulations of coral reef freshwater TA endmembers (TA_S = 0_ ± uncertainties) to approximate the potential uncertainties associated with the salinity normalization of coral reef TA data. We conclude that salinity normalization can change both the sign and magnitude of ΔnTA across coral reef environments suggesting that future studies should exercise caution when evaluating and interpreting ΔnTA from coral reef TA and S data. This is especially true for studies with larger ranges in salinity because the uncertainties associated with ΔnTA positively scale with changes in S. We offer several considerations for salinity normalizing coral reef carbonate chemistry data in the future to reduce potential ΔnTA uncertainties when using the alkalinity anomaly technique as a proxy for net coral reef calcification in any reef system:

1. Do not automatically assume salinity normalization is necessary, as it could introduce more error into your measurements than expected.
2. Examine data for any detectable correlations between salinity and TA as these relationships indicate the potential influence of salinity dilution or concentration mechanisms on TA. Even if there are no detectable correlations between salinity and TA, evaluate the potential role that simple dilution or concentration mechanisms may have on coral reef TA data.
3. Precisely measure both salinity and TA. Consider the range and precision of your salinity measurements and the potential impact that S normalization has on TA values. If the range in salinity is within the precision of the salinity measurement, consider not salinity normalizing your data.
4. Take a holistic approach to examine possible sources and relative contributions of freshwater and submarine groundwater discharge to the reef system. Consider taking direct freshwater samples to better determine potential endmembers (i.e., TA_S = 0_) that are mixing with the reef water mass to reduce ΔnTA uncertainties and consider avoiding reef metabolism studies in regions with large salinity changes that may confound conclusions regarding ΔnTA.
5. When the relative volumetric contributions of multiple TA_S = 0_ are unknown, consider calculating ΔnTA for a range of TA_S = 0_ values to propagate the potential ΔnTA ± uncertainties owing to unknown TA_S = 0_.
6. Carefully select S_ref_ while considering the implicit order of mixing and biogeochemical modification. The mean and range of ΔnTA_offshore_ (i.e., S_ref_ = S_offshore_) and ΔnTA_reef_ (i.e., S_ref_ = S_reef_) can be used to estimate the ΔnTA ± uncertainties owing to the implicitly assumed order of mixing in the salinity normalization of TA.
7. Propagate the potential uncertainties associated with the salinity normalization of coral reef TA data on calculated ΔnTA to evaluate the relative importance of the salinity normalization process to the resulting estimate of ΔnTA and calculated reef metabolism.

These findings build upon our previous assessment that depth and residence time are the primary sources of NEC uncertainty [17] to include the potentially large uncertainties associated with salinity normalization of TA and DIC data. While this study has focused on coral reef TA for the purpose of brevity, many of these same findings also apply to the salinity normalization of dissolved inorganic carbon (DIC) and other ions of interest (e.g., Ca^2+^, Mg^2+^) for coral reef metabolism studies, which could be exacerbated depending on their background concentrations in seawater. Reducing uncertainties in the evaluation of coral reef metabolism (e.g. calcification, dissolution, respiration, and photosynthesis) remains critical for observing and quantifying coral reef function and health under unprecedented environmental change.

## Supporting information

S1 Table Summary table of coral reef total alkalinity and salinity. *Source* refers to the following reef locations: 01 = Makapu’u, 03 = Palmyra, 04 = Red Sea 1997, 05 = Red Sea 1998, 06 = Red Sea 2013, 07 = Ofu, 08 = Heron Island, 09 = Palau 1, 11 = Lizard Island, 12 = Ishigaki, 13 = One Tree 1, 14 = Kaiona, 15 = Lady Elliot, 16 = One Tree 2, 17 = Davies, 18 = Palau 2, 19 = Cook Islands, 20 = Cheeca Rocks, 21 = St. John, 22 = West Panama, 23 = Florida Keys, 24 = Bermuda, 25 = Majuro, 26 = Maldives, and 27 = Puerto Rico sensu [2], *method* refers to OLS = ordinary least squares regression, *term* represents the Slope of the relationship, *estimate* represents the value of the slope, *conf*.*low* represents the lower 95% confidence interval of the slope, *conf*.*high* represents the upper 95% confidence interval of the slope, and *p*.*value* represents the p-value of the slope.
(CSV)
